# High prevalence of hepatitis B and poor knowledge on hepatitis B and C viral infections among barbers: a cross-sectional study of the Obuasi municipality, Ghana

**DOI:** 10.1186/s12889-015-2389-7

**Published:** 2015-10-11

**Authors:** Prince Adoba, Stephen Kyei Boadu, Hope Agbodzakey, Daniel Somuah, Richard Kobina Dadzie Ephraim, Enoch Anto Odame

**Affiliations:** Medical Laboratory Division, Department of Laboratory Technology, University of Cape Coast, Cape Coast, Ghana; Laboratory Department, Edwin Cade Memorial Hospital, Obuasi, Ghana; Department of Molecular Medicine, School of Medical Sciences, College of Health Sciences, Kwame Nkrumah University of Science and Technology, Kumasi, Ghana

**Keywords:** Hepatitis B virus (HBV), Hepatitis C virus (HCV), Barbers, Knowledge

## Abstract

**Background:**

Barbers, while shaving, may be accidentally exposed to the blood and bodily fluids of their customers increasing their risk of contraction of HBV and HCV infections. Hence, this study aimed at examining the prevalence and knowledge of barbers on HBV and HCV infections in the Obuasi municipality of Ghana.

**Methods:**

A work place based cross-sectional study was conducted from January to April 2015 at the Obuasi municipality in the Ashanti Region of Ghana. Two hundred (200) barbers were conveniently recruited and blood sample of each participant collected for the detection of HBsAg and HCV antibodies. Data on socio demographic characteristics, and knowledge on HBV and HCV infections were collected using a structured and pre-tested questionnaire. Analysis was performed using SPSS version 16.0, and P < 0.05 was considered statistically significant.

**Results:**

The prevalence of HBV and HCV among the barbers were 14.5 % and 0.5 % respectively. HBV was highest among barbers within 20–29 years (58.6 %). Majority (90.5 %) of the participants had heard of HBV infection before. The mode of transmission of HBV was unknown by 64.5 % of the participants and 64.0 % did not perceive themselves to be at risk for HBV. Most of the participants had never heard of HCV infection (61.3 %), and unaware of any mode of transmission of HCV (97.0 %). The radio was the major source of information on HBV (57.5 %) and HCV (25.0 %) infections.

**Conclusion:**

High prevalence of HBV and low knowledge on HBV and HCV infections was found among barbers. Barbers need to be educated on viral hepatitis to reduce the acquisition of HBV and HCV infections.

## Background

Hepatitis B (HBV) and Hepatitis C (HCV) viral infection are common chronic blood-borne infectious diseases affecting two billion and 3.9 million people respectively globally, including an estimated 400 million chronically infected with HBV [1]. Infection by HBV and HCV cause serious mortality and morbidity, and are thus a major global health problem. A significant proportion of those exposed to HBV become chronically infected and are at considerable risk of liver cancer, chronic active hepatitis and cirrhosis. These infected people may not be aware of their HBV status and are not clinically ill but are a source of infection to others [2]. Transmission can be through bodily secretions such as saliva, sweat, urine as well as blood and blood products. Barbers while shaving may be accidentally exposed to the blood and bodily fluids of their customers [3]. In many parts of Africa, the widespread cultural practice of shaving at barbershops or from a roadside barber might be a route of blood-borne viral disease transmission. A study conducted by Shalaby *et al*. [4] found a 4.1 % HBV prevalence and 12.5 % HCV prevalence rate among barbers in Gharbia governorate, Egypt. Belbacha *et al*. [5] also observed a 28 % and 1.1 % prevalence rates of HBV and HCV respectively among traditional barbers in the Rabat region of Morocco. Abbasi *et al*. [6] reported a 2.1 % prevalence of HBV among barbers in a cross-sectional survey conducted in the district of Sukkur of the Sindh Province in Pakistan, and Candan *et al*. [3] also found a higher prevalence of HBV and HCV among barbers (39.8 % and 2.8 % respectively) than the comparison group (28.3 % and 1.1 % respectively) in a study conducted in the Sivas region of Turkey. However, despite this high occupational hazard posed to barbers, several studies have reported low level of knowledge on HBV and HCV among barbers in developing countries [7–9]. Also, data on the prevalence, knowledge and awareness of HBV and HCV among barbers are lacking in Ghana, and sub-Saharan Africa. This study, hence, sought to examine the prevalence and knowledge on HBV and HCV infections among barbers in the Obuasi municipality of Ghana.

## Methods

### Study design/study site

This work place based cross-sectional study was conducted from January to April 2015 at the Obuasi municipality in the Ashanti region of Ghana. Obuasi is a major gold mining town in Ghana, with its inhabitants being mainly miners. The municipality has a population of 168, 641 [10] and is the second largest district in the Ashanti Region.

### Study population

Two hundred (200) barbers working in the municipality were conveniently recruited onto the study. In order to determine the required sample size, the formula: n = Z^2^PQ/d^2^ was used, where, Z = 1.96, P = prevalence of hepatitis B from a previous study i.e. 0.082; Q = 1-P i.e. 0.918 and d = margin of error i.e. 0.05. Thus, the calculated sample size was n = 115. With the minimum number to be enrolled being 115, we used 200 individuals in this study. Data on socio demographic characteristics, and knowledge on HBV and HCV infections of all barbers who were willing and consented to participate in the study were collected by face to face interview using a structured and pre-tested questionnaire before taking their blood samples. Participants with history of HBV and HCV infections prior to the start of the profession were excluded.

### Ethical consent

The study was approved by the University of Cape Coast Institutional Review Board (UCCIRB), and informed written consent sought from the participants before collecting their data and samples.

### Blood sample collection

About 3 ml of venous blood was drawn from each study participant, dispensed into a serum separator tube and transported on ice to the laboratory. At the laboratory, the samples were centrifuged at 1500 rpm for 5–10 minutes to obtain serum for serological testing.

### Serological tests

The serum was screened for HBsAg using commercially available rapid test strips (Abon Biopharm Co. Ltd, Hangzhou, China), and anti-HCV antibodies using Global HCV-Ab test strip (Global Diagnostics, USA).

### Statistical analysis

Data were stored in Microsoft Excel and analyzed using Statistical Package for Social sciences (SPSS) version 16.0 software. Descriptive analysis was performed and the results expressed as numbers and percentages. Multivariable logistic regression was used to determine the likelihood of HBV and HCV infections. *P* < 0.05 was considered statistically significant.

## Results

Majority of the participants (57.5 %) were within the ages of 20–29 years, single (71.0 %), had secondary education (52.5 %), and worked as barbers for 1–5 years (49.0 %). Most of them had never received blood transfusion (98.5 %), never undergone dental surgery (91.0 %), had no tattoo or body piercing (85.0 %), and had never used intravenous drugs (92.0 %). On the other hand, a significant number had had unprotected sex before (84.5 %). Out of the 200 participants screened, (14.5 %) were positive for HBV and 1 (0.5 %) was positive for HCV (Table 1).

**Table 1:** General characteristics of study participants

Variable	*N* (%)
*Age* (*years*)	28.18 ± 7.04
*Age group*	
<20	11 (5.5)
20-29	115 (57.5)
30-39	56 (28.0)
40-49	18 (9.0)
*Marital status*	
Single	142 (71.0)
Married	56 (28.0)
Divorced	2 (1.0)
*Educational level*	
None	7 (3.5)
Basic	76 (38.0)
Secondary	105 (52.5)
Tertiary	12 (6.0)
*Working Experience* (*years*)	
<1	7 (3.5)
1-5	98 (49.0)
6-10	54 (27.0)
11-15	20 (10.0)
16-20	15 (7.5)
>20	6 (3.0)
*Ever had Blood transfusion*?	
Yes	3 (1.5)
No	197 (98.5)
*Ever undergone dental procedure*?	
Yes	18 (9.0)
No	182 (91.0)
*Tattoo*/*Body piercing*	
Yes	30 (15.0)
No	170 (85.0)
*Use of IV drugs*	
Yes	16 (8.0)
No	184 (92.0)
*Ever had unprotected sex before*?	
Yes	169 (84.5)
No	31 (15.5)
*HBV Status*	
Positive	29 (14.5)
Negative	171 (85.5)
*HCV Status*	
Positive	1 (0.5)
Negative	199 (99.5)

Table 2 shows the socio-demographic and clinical characteristics of study participants in relation to HBV status. Majority of the HBV-positive participants were within the ages 20–29 (58.6 %), single (79.3 %), and had secondary education (55.2 %). The highest incidence of HBV was found in participants with 1–5 years working experience (51.7 %). All the participants with HBV had not received blood transfusion before (100 %), never undergone a dental procedure (96.6 %), not tattooed or had body piercing (89.7 %), and not used IV drugs before (86.2 %). On the contrary, majority (82.8 %) of those infected with HBV had had unprotected sex before. Age, marital status, educational level and years of working were not associated with HBV status (*P* > 0.05).

**Table 2:** Socio-demographic and clinical characteristics of study participants in relation to HBV status

Variable	HBV status		*P*-value
	Positive	Negative	
	(N = 29)	(N = 171)	
*Age group*			0.211
<20	1 (3.4)	10 (5.8)	
20-29	17 (58.6)	98 (57.3)	
30-39	11 (37.9)	45 (26.3)	
40-49	0 (0.0)	18 (10.5)	
*Marital status*			0.518
Single	23 (79.3)	119 (69.6)	
Married	6 (20.7)	50 (29.2)	
Divorced	0 (0.0)	2 (1.2)	
*Educational level*			0.977
None	1 (3.4)	6 (3.5)	
Basic	10 (34.5)	66 (38.6)	
Secondary	16 (55.2)	89 (52.0)	
Tertiary	2 (6.9)	10 (5.8)	
*Working Experience* (*years*)			0.423
<1	2 (6.9)	5 (2.9)	
1-5	15 (51.7)	83 (48.5)	
6-10	9 (31.0)	45 (26.3)	
11-15	3 (10.3)	17 (9.9)	
16-20	0 (0.0)	15 (8.8)	
>20	0 (0.0)	6 (3.5)	
*Ever had Blood transfusion*?			0.472
Yes	0 (0.0)	3 (1.8)	
No	29 (100)	168 (98.2)	
*Ever undergone dental procedure*?			0.259
Yes	1 (3.4)	17 (9.9)	
No	28 (96.6)	154 (90.1)	
*Tattoo*/*Body piercing*			0.448
Yes	3 (10.3)	27 (15.8)	
No	26 (89.7)	144 (84.2)	
*Use of IV drugs*			0.214
Yes	4 (13.8)	12 (7.0)	
No	25 (86.2)	159 (93.0)	
*Ever had unprotected sex before*?			0.779
Yes	24 (82.8)	145 (84.8)	
No	5 (17.2)	26 (15.2)	
*Vaccinated for Hepatitis B*			0.182
Yes	0 (0.0)	10 (5.8)	
No	29 (100)	161 (94.2)	

The socio-demographic and clinical characteristics of study participants stratified by HCV status is presented in Table 3. The participant positive for HCV was within the ages of 30–39, married with 1–5 years working experience. He had never received blood transfusion or undergone a dental procedure, but had had unprotected sex previously.

**Table 3:** Socio-demographic characteristics of study participants stratified by HCV status

Variable	HCV status	
	Positive	Negative
	(*N* = 1)	(*N* = 199)
*Age group*		
<20	0 (0.0)	11 (5.5)
20-29	0 (0.0)	115 (57.8)
30-39	1 (100)	55 (27.6)
40-49	0 (0.0)	18 (9.0)
*Marital status*		
Single	0 (0.0)	142 (71.4)
Married	1 (100)	55 (27.6)
Divorced	0 (0.0)	2 (1.0)
*Educational level*		
None	0 (0.0)	7 (3.5)
Basic	1 (100)	75 (37.7)
Secondary	0 (0.0)	105 (52.8)
Tertiary	0 (0.0)	12 (6.0)
*Working Experience* (*years*)		
<1	0 (0.0)	7 (3.5)
1-5	0 (0.0)	98 (49.2)
6-10	0 (0.0)	54 (27.1)
11-15	0 (0.0)	20 (10.1)
16-20	1 (100)	14 (7.0)
>20	0 (0.0)	6 (3.0)
*Ever had Blood transfusion*?		
Yes	0 (0.0)	3 (1.5)
No	1 (100)	196 (98.5)
*Ever undergone dental procedure*?		
Yes	0 (0.0)	18 (9.0)
No	1 (100)	181 (91.0)
*Tattoo*/*Body piercing*		
Yes	0 (0.0)	30 (15.1)
No	1 (100)	169 (84.9)
*Use of IV drugs*		
Yes	0 (0.0)	16 (8.0)
No	1 (100)	183 (82.0)
*Ever had unprotected sex before*?		
Yes	1 (100)	168 (84.4)
No	0 (0.0)	31 (15.6)

Table 4 describes the multivariable logistic regression analysis of factors associated with HBV and HCV infection among the barbers. Barbers who were single and those in their third and fourth decades of life were insignificantly more likely to have HBV infection (*P* > 0.05).

**Table 4:** Multivariable logistic regression analysis of factors associated with HBV infection among barbers

Variable	OR (95 % CI)	*P*-value
*Age group*		
<20	Referent	
20-29	1.74 (0.21-14.44)	0.610
30-39	2.5 (0.29-21.66)	0.406
*Marital status*		
Single	1.61 (0.62 to 4.19)	0.319
Married	Referent	
*Educational level*		
None	Referent	
Basic	0.92 (0.1-8.49)	0.944
Secondary	1.08 (0.12-9.57)	0.946
Tertiary	1.20 (0.09-16.24)	0.891
*Working Experience* (*years*)		
<1	Referent	
1- 5	0.45 (0.08-2.55)	0.368
6- 10	0.50 (0.08-2.99)	0.448
11- 15	0.44 (0.06-3.42)	0.434
*Ever had blood transfusion*?		
Yes	1.22 (0.06-24.30)	0.996
No	Referent	
*Ever undergone dental procedure*?		
Yes	0.35 (0.04-2.76)	0.279
No	Referent	
*Tattoo*/*Body piercing*		
Yes	0.62 (0.17-2.16)	0.580
No	Referent	
*Use of IV drugs*		
Yes	2.01 (0.63-7.05)	0.227
No	Referent	
*Ever had unprotected sex before*?		
Yes	0.87 (0.30-2.48)	0.788
No	Referent	

Knowledge on HBV among the participants in relation to HBV status is described in Table 5. Majority (90.5 %) of the participants had heard of HBV infection before, with the radio being the major source of information (57.5 %). The mode of transmission of HBV was unknown by 64.5 % of the participants and 64.0 % did not perceive themselves to be at risk for HBV. None of the HBV-positive participants knew that reuse of needles and barber shaving instruments could transmit HBV infection.

**Table 5:** Knowledge on HBV among the participants in relation to HBV status

Variable	Total	HBV status		*P*-value
		Positive	Negative	
	(*N* = 200)	(*N* = 29)	(*N* = 171)	
*Have you ever heard about viral hepatitis B*?				0.394
Yes	181 (90.5)	25 (86.2)	156 (91.2)	
No	19 (9.5)	4 (13.8)	15 (8.8)	
*Source of information on HBV*				
Friends and relatives	11 (5.5)	1 (3.4)	10 (5.8)	0.600
Television	39 (19.5)	7 (24.1)	32 (18.7)	0.495
Newspapers	9 (4.5)	0 (0.0)	9 (5.3)	0.206
Radio	115 (57.5)	16 (55.2)	99 (57.9)	0.784
Healthcare workers	20 (10.0)	3 (10.3)	17 (9.9)	0.947
*Mode of transmission of HBV*				0.052
Blood transfusion	2 (1.0)	1 (3.4)	1 (0.6)	
Reusing needles	2 (1.0)	0 (0.0)	2 (1.2)	
Body contact	19 (9.5)	5 (17.2)	14 (8.2)	
Droplets	23 (11.5)	0 (0.0)	23 (13.5)	
Barbers shaving instruments	1 (0.5)	0 (0.0)	1 (0.6)	
Sexual contact	22 (11.0)	6 (20.7)	16 (9.4)	
Tattooing	2 (1.0)	1 (3.4)	1 (0.6)	
Don’t know	129 (64.5)	16 (55.2)	113 (66.1)	
*Do you perceive yourself to be at risk for hepatitis B*?				0.854
Yes	72 (36.0)	10 (34.5)	62 (36.3)	
No	128 (64.0)	19 (65.5)	109 (63.7)	

Knowledge on HCV among the participants in relation to HCV status is shown in Table 6. Most of the participants had never heard of HCV infection (61.3 %), were unaware of any mode of transmission of HCV (97.0 %) and did not perceive themselves to be at risk for HCV (64.0 %). The radio was the major source of information on HCV infection (25.0 %).

**Table 6:** Knowledge on HCV among the participants in relation to HCV status

Variable	Total	HCV status	
		Positive	Negative
*Have you ever heard about viral hepatitis C*?			
Yes	78 (39.0)	1 (100)	77 (38.7)
No	122 (61.3)	0 (0.0)	122 (61.3)
*Source of information on HCV*			
Friends and relatives	7 (3.5)	0 (0.0)	7 (3.5)
Television	19 (9.5)	0 (0.0)	19 (9.5)
Newspapers	1 (0.5)	0 (0.0)	1 (100)
Radio	50 (25.0)	1 (100)	49 (24.6)
Healthcare workers	9 (4.5)	0 (0.0)	9 (4.5)
*Mode of transmission of HCV*			
Blood transfusion	0 (0.0)	0 (0.0)	0 (0.0)
Reusing needles	0 (0.0)	0 (0.0)	0 (0.0)
Body contact	0 (0.0)	0 (0.0)	0 (0.0)
Droplets	0 (0.0)	0 (0.0)	0 (0.0)
Barbers shaving instruments	0 (0.0)	0 (0.0)	0 (0.0)
Sexual contact	6 (3.0)	0 (0.0)	6 (3.0)
Tattooing	0 (0.0)	0 (0.0)	0 (0.0)
Don’t know	194 (97.0)	1 (100)	193 (97.0)
*Do you perceive yourself to be at risk for hepatitis C*?			
Yes	72 (36.0)	1 (100)	71 (35.7)
No	128 (64.0)	0 (0.0)	128 (64.3)

## Discussion

Many traditional and low-paid barbers in developing countries earn their livelihood by providing shaving services in the marketplace and on the street side [11]. Poor infection control during the use of sharp instruments may be a risk factor for blood-borne infections, potentially causing serious health problems for both the barber and the clients [12]. This cross-sectional study examined the prevalence of Hepatitis B viral (HBV) infection and hepatitis C viral (HCV) infections among barbers in the Obuasi municipality of Ghana. The prevalence of HBV and HCV among the barbers were 14.5 % and 0.5 % respectively. Most of the participants had low level of knowledge on HBV and HCV infections.

The 14.5 % prevalence of HBV obtained in this study is higher than the 4.1 % prevalence recorded in a cross-sectional study by Shalaby *et al*. [4] to determine the prevalence of both HBV and HCV infections among barbers and a sample of their clients in Gharbia governorate, Egypt. However, it is lower than the 28 % recorded by Belbacha *et al*. [5] in a cross-sectional epidemiological study to determine the prevalence of HBV and HCV among traditional barbers and their clients in the Rabat region of Morocco. The difference in prevalence rates could be due to the differences in sample sizes, geographical location and sampling techniques used. It is also higher than the 8.2 % HBV prevalence reported among blood donors in 2012 by Walana *et al*. [13] in a retrospective hospital-based study conducted at Kintampo municipal in Ghana, and the 8.68 % prevalence observed by Amidu *et al*. [14] in a prospective study conducted at Kumasi in Ghana. This shows a high prevalence rate of HBV among barbers compared to the general population.

The 0.5 % prevalence of HCV recorded is, however, lower than the 1.1 % prevalence rate reported by Belbacha *et al*. [5] in Morocco and the 12.5 % prevalence rate reported by Shalaby *et al*. [4] in Egypt. The significantly lower prevalence of HCV found in this study compared to the findings of Shalaby *et al*. might be due to the differences in sample size, and the higher endemicity of HCV in Egypt [4]. It is also lower than the 2.3 % HCV prevalence reported among blood donors in 2012 by Walana *et al*. [13] in a retrospective hospital-based study conducted at Kintampo municipal in Ghana, indicating a low HCV infection rate among barbers.

Dongdem and colleagues [15] in a cross-sectional study to estimate the prevalence of HBV among blood donors at the Tamale Teaching Hospital in Ghana observed the highest prevalence of HBV among donors within the ages of 20–29. This supports the finding of the highest prevalence of HBV among barbers within the ages of 20–29 years in our study. The high prevalence of HBV infection among the youth could be the result of risky lifestyles [15, 16].

The finding of no significant association between the risk factors and HBV infection among the barbers in the multivariable logistic regression indicates a probable high occupational risk of barbers to HBV infection. However, this assertion should be done with caution since this study did not assess a causal relationship between HBV and barbering.

Studies carried out in Morocco, Ethiopia and Pakistan showed that the level of knowledge and awareness of barbers about the concept of infectious risk associated with blood was generally very low [17, 18]. This is similar to the finding of majority of the study participants not being aware of the HCV infection, and the modes of transmission of HBV and HCV. It is also in line with the study by Belbacha *et al*. [5] which found that traditional barbers and their clients in Morocco are unfamiliar with HBV and HCV and are mostly unaware of the transmission of blood borne pathogens through shaving tools.

Majority of the participants did not perceive themselves to be at risk of HBV and HCV infections. This is in line with the observations of Wazir *et al*. [19] in which the level of knowledge among barbers about health hazards associated with their profession was found to be very poor, in a descriptive cross-sectional study to assess awareness among barbers regarding health hazards related to their profession and to identify professional practices linked with infection transmission in Pakistan.

Micro-trauma induced while shaving cause release of blood and other bodily fluids which can cause transmission of HBV and HCV to barbers when they come in contact. Also, contamination of the shaving instruments can pose a great risk to other clients. Despite this, few barbers were aware of the risk posed by unsafe shaving practices and the mode of transmission of HBV and HCV infections. For these reasons, awareness campaigns are imperative and should be focused on both barbers and the general population especially those who are at risk due to their occupation. Also, HBV vaccination should be encouraged in order to curb the increasing incidence of HBV.

The study is limited by the smaller sample size, and the inability to use more sensitive and specific diagnostic methods like polymerase chain reaction (PCR) due to lack of resources. A causal relationship between barbering and HBV and HCV infections could not be assessed due to the cross-sectional nature of the study.

## Conclusion

HBV and HCV was prevalent in 14.5 % and 0.5 % of the barbers respectively. The highest prevalence of HBV was found among barbers in their third decade of life., with barbers who are single being more likely to acquire HBV infection. Most of the study participants were not aware of the HCV infection, and the mode of transmission of both HBV and HCV infections. There is, therefore, the need to educate both the barbers and their clientele about viral hepatitis to reduce the acquisition of hepatitis B and C infections at barbershops. Also, mechanisms should be put in place to ensure registration and compliance of barbers with occupational safety regulations.

## Abbreviations

HBV: Hepatitis B virus
HCV: Hepatitis C virus
